# Display of Cell Surface Sites for Fibronectin Assembly Is Modulated by Cell Adherence to ^1^F3 and C-Terminal Modules of Fibronectin

**DOI:** 10.1371/journal.pone.0004113

**Published:** 2009-01-01

**Authors:** Jielin Xu, Eunnyung Bae, Qinghong Zhang, Douglas S. Annis, Harold P. Erickson, Deane F. Mosher

**Affiliations:** 1 Department of Biomolecular Chemistry, University of Wisconsin-Madison, Madison, Wisconsin, United States of America; 2 Department of Pathology and Laboratory Medicine, University of Wisconsin-Madison, Madison, Wisconsin, United States of America; 3 Department of Medicine, University of Wisconsin-Madison, Madison, Wisconsin, United States of America; 4 Department of Cell Biology, Duke University Medical Center, Durham, North Carolina, United States of America; University of Giessen Lung Center, Germany

## Abstract

**Background:**

Fibronectin-null cells assemble soluble fibronectin shortly after adherence to a substrate coated with intact fibronectin but not when adherent to the cell-binding domain of fibronectin (modules ^7^F3-^10^F3). Interactions of adherent cells with regions of adsorbed fibronectin other than modules ^7^F3-^10^F3, therefore, are required for early display of the cell surface sites that initiate and direct fibronectin assembly.

**Methodology/Principal Findings:**

To identify these regions, coatings of proteolytically derived or recombinant pieces of fibronectin containing modules in addition to ^7^F3-^10^F3 were tested for effects on fibronectin assembly by adherent fibronectin-null fibroblasts. Pieces as large as one comprising modules ^2^F3-^14^F3, which include the heparin-binding and cell adhesion domains, were not effective in supporting fibronectin assembly. Addition of module ^1^F3 or the C-terminal modules to modules ^2^F3-^14^F3 resulted in some activity, and addition of both ^1^F3 and the C-terminal modules resulted in a construct, ^1^F3-C, that best mimicked the activity of a coating of intact fibronectin. Constructs ^1^F3-C V0, ^1^F3-C V64, and ^1^F3-C Δ(V^15^F3^10^F1) were all able to support fibronectin assembly, suggesting that ^1^F3 through ^11^F1 and/or ^12^F1 were important for activity. Coatings in which the active parts of ^1^F3-C were present in different proteins were much less active than intact ^1^F3-C.

**Conclusions:**

These results suggest that ^1^F3 acts together with C-terminal modules to induce display of fibronectin assembly sites on adherent cells.

## Introduction

**Figure 1:**
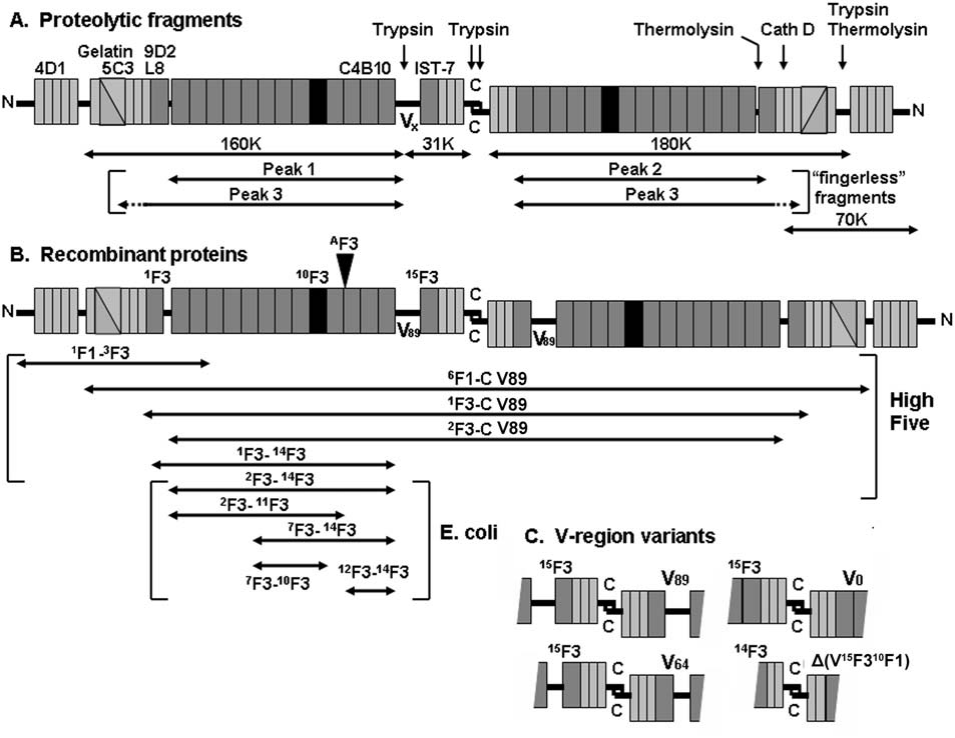
Diagram of proteolytic fragments, recombinant proteins, location of antigenic epitopes, and relevant features of fibronectin. (A) Diagram of modular make-up of plasma fibronectin, proteolytic fragments, and locations of antigenic epitopes and sites of proteolytic cleavage. N-termini (N) are on the outsides, and C-termini (C) are in the middle. The bulk of each subunit consists of 12 type I (thin rectangles), two type II (triangles), and 15 type III modules (thick rectangles). The subunits are joined near the C-termini by a pair of disulfide bridges. Sites of proteolytic cleavage are shown for the subunit on the right (Cath D, cathepsin D). The subunit to the left contains the alternatively spliced V region that is susceptible to trypsin. Locations of antigenic epitopes and gelatin-binding activity are shown for the subunit on the left. Monomeric fragments generated uniquely from the two subunits by trypsin, without or with (“fingerless” fragments) prior reduction and alkylation, are shown below the subunits. Fragments generated from both subunits are shown only on the right. (B) Diagram of recombinant pieces of fibronectin. The layout is as described in panel A. Key F3 modules are indicated for the subunit on the left. These proteins were made without or with ^A^F3 inserted between ^11^F3 and ^12^F3 (arrowhead), as described in the text. The pieces shown were generated using cDNA encoding subunits with the V89 version of the V region and no ^B^F3. Modular compositions of the various recombinant proteins produced in High Five cells or E. coli are shown below. Recombinant proteins containing the C-terminus form dimers (such as ^1^F3-C) and recombinant proteins without the C-terminus were expressed as monomers (such as ^1^F3-^14^F3). (C) Diagram of the different alternatively spliced forms of the V region that we expressed in ^1^F3-C dimers.

Fibronectin is a dimer of nearly identical subunits composed largely of types I (F1), II (F2), and III (F3) fibronectin modules (Fig. 1A). Fibronectin is present as an abundant soluble protein in blood plasma and an insoluble extracellular matrix (ECM) protein. Assembly of fibronectin into ECM is a cell-dependent process that is initiated at specialized sites on cell surfaces [1]. Display of these sites requires active integrins [2]–[10]. The “handle” in soluble fibronectin that cells use to initiate assembly, however, is located in the N-terminal F1 and F2 modules, not the RGD-containing F3 module recognized by α5β1 and several other integrins [11].

Assembly of exogenously added soluble plasma fibronectin by adherent fibronectin-null cells is dependent upon the substrate to which the cells are attached; cells adherent to surface-adsorbed fibronectin or laminin are competent to assemble exogenous fibronectin whereas cells adherent to surface-adsorbed vitronectin or the cell-binding domain of fibronectin (^7^F3-^10^F3), which contains the RGD sequence recognized by integrins, assemble exogenous fibronectin poorly [9], [12]–[14]. A similar difference is true for binding of the N-terminal 70-kDa fragment of fibronectin that binds to the surface sites that initiate fibronectin assembly [11]; fibronectin-null cells adherent to surface-adsorbed fibronectin or laminin-1 bind 70-kDa fragment whereas cells adherent to surface-adsorbed vitronectin do not [11]. Co-coating experiments demonstrated that the presence of vitronectin on a surface suppresses the ability of surface-adsorbed fibronectin or laminin to support fibronectin assembly by adherent fibronectin-null cells whereas ^7^F3-^10^F3 lacks such suppressive activity [12]. These results indicate that the interaction of adherent cells with regions of adsorbed fibronectin other than the RGD-containing integrin modules recognized by α5β1 is needed to support initiation of fibronectin assembly. Such regions could include the V (CS) region or ^4^F3-^5^F3 recognized by α4β1 integrin [15], [16], the ^12^F3-^14^F3 modules (HepII domain) that bind syndecans [17], the gelatin-binding domain that binds to cellular (type 2) transglutaminase [18], [19], the GNGR sequences in type I modules that have the potential to isomerize to G*iso*DGR recognized by αvβ3 [10], [20], or the IGD sequences in type I modules important for the motogenic activity of fibroblasts [21]. Assembly of fibronectin by cellular fibronectin-expressing cells is less sensitive to the nature of the adherent substrate [12]. One explanation for the insensitivity is that newly secreted endogenous fibronectin mimics activities of substrate-coated fibronectin [11].

To search for the hypothesized supportive region(s) in fibronectin, sets of proteolytically generated or recombinant pieces of fibronectin were tested. Surprisingly, pieces as large as one comprising modules ^2^F3-^14^F3, which contain the heparin-binding and cell adhesion domains, were only minimally active in supporting fibronectin assembly. In contrast, pieces in which the 1^st^ type III module (^1^F3) and C-terminal modules (including V region, ^15^F3, and ^10^F1-^12^F1) flanked ^2^F3-^14^F3 (see Fig. 1B) were nearly as active as full-length fibronectin.

## Materials and Methods

### Cells

Fibronectin −/− (fibronectin-null) fibroblastic cells from fibronectin −/− mouse embryonic stem cells were derived and handled as described previously [12], [22].

### Preparation of adhesive substrates and cells for experiments

The adhesive proteins were usually diluted to 20 nM in phosphate buffered saline, pH 7.4 (PBS) prior to coating overnight at 4°C of glass cover slips or wells of 24-well tissue culture plates. The surfaces were then coated with 1% fatty acid-poor bovine serum albumin (BSA, Sigma, St. Louis, MO) for 40 min at 37°C and washed with PBS. Cells, which had been suspended from confluent monolayers by trypsinization followed by inhibition of trypsin with fetal calf serum and repeated centrifugation alternating with suspension in PBS to remove serum proteins, were seeded in the coated wells at 37°C in Dulbecco's modification of Eagle's medium (DMEM, Cellgro Mediatech, VA) supplemented with 0.2% fatty acid-poor BSA. The number of cells added was determined in pilot experiments to result in monolayers of spread cells 60∼70% confluent after 4 hr.

### Preparation of fibronectin and proteolytic fragments of fibronectin

Human fibronectin was purified from a fibronectin-rich side fraction of plasma processing by precipitation of contaminating fibrinogen by brief heating at 56°C followed by chromatography on DEAE-cellulose [23].

A schematic of the fibronectin proteolytic fragments and recombinant constructs are shown in Fig. 1.

“Fingerless” fibronectin was prepared by reduction and alkylation, limited trypsin treatment, and gel filtration [24]. The method is based on the finding that disulfide modification greatly increases the susceptibility of N- and C-terminal type I and II modules to trypsinization [25]. A mixture of fragments was obtained [24]. This mixture was dialyzed against buffer A (25 mM Tris pH 7.4, 50 mM NaCl) and applied to an anionic exchange column (monoQ HR 5/5, GE Healthcare, Piscataway, NJ) in an Akta FPLC (GE Healthcare). Proteins were separated by linear gradient of buffer B (25 mM Tris pH 7.4, 1 M NaCl) and characterized by immunoblotting with previously characterized monoclonal antibodies L8, 9D2, C4B10, or IST-7 [26] or antibodies 4D1 or 5C3 generated at the University of Alabama-Birmingham [27]. For the immunoblotting, peroxidase-conjugated goat anti-mouse IgG (H+L) (Jackson ImmunoResearch, West Grove, PA) was used as a secondary antibody.

Gelatin-binding 160/180-kDa fibronectin fragments were purified from a limited tryptic digestion of native plasma-derived fibronectin [28]. The C-terminal 31-kDa fragment was purified from the non-gelatin-binding fraction of the same limited tryptic digest [29]. The N-terminal 70-kDa catheptic D fragment of fibronectin was purified as described previously [24].

### Recombinant proteins

Proteins are named according to their N- and C-termini with the nomenclature of Campbell *et. al.* [30]. Also included in the name are “A” if ^A^F3 was present and the version of the V-region [31]–[35].

Bacterially synthesized recombinant proteins ^7^F3-^10^F3, ^12^F3-^14^F3, ^7^F3-^14^F3, ^7^F3-^14^F3A, ^2^F3-^11^F3, and ^2^F3-^14^F3 (Fig. 1B) were purified and characterized as described [9], [36], [37].

Recombinant proteins ^1^F1-^3^F3, ^6^F1-C V89, ^6^F1-CA V89, ^1^F3-C V89, ^1^F3-CA V89, ^1^F3-^14^F3, ^2^F3-C V89, ^1^F3-C V0, ^1^F3-CA V0, ^1^F3-C V64, ^1^F3-CA V64, ^1^F3-C Δ(V^15^F3^10^F1), and ^1^F3-CA Δ(V^15^F3^10^F1) (Fig. 1B) were produced using a baculovirus expression system [38]. Recombinant baculovirus was generated by co-transfection of monolayers of SF9 cells (Invitrogen, Carlsbad, CA) with BaculoGold linearized AcNPV viral DNA (BD Biosciences, San Jose, CA) and the appropriate transfer vector using CellFectin (Invitrogen). The transfer vectors were constructed by cloning PCR-amplified pieces of DNA of human fibronectin into pCOCO; pCOCO endows the recombinant protein with an N-terminal signal peptide and C-terminal polyhistidine tag [38]. Viruses from individual plaques were amplified, and used after pass 3 to infect High Five insect cells (Invitrogen) in SF900II serum-free medium at 27°C. Sodium azide, 0.1%, and polymethylsulfonyl fluoride, 2 mM, were added to conditioned medium collected ∼65 hours post infection. The medium was dialyzed against TBS (10 mM Tris, pH 7.4, 130 mM NaCl) and then incubated with a suspension of Ni^2+^-nitriloacetic acid resin (Qiagen, Valencia, CA) overnight at room temperature. The resin was collected by centrifugation, placed in a small column, and washed with TBS buffer containing 15 mM imidazole. Polyhistidine-bearing protein was eluted with TBS buffer containing 300 mM imidazole, and the purified protein, 200–1000 µg/ml, was dialyzed against Tris buffer containing 1 M sodium bromide, and stored in portions at −80°C until used. Alternatively, MOPS buffer (10 mM MOPS, pH 7.4, 300 mM NaCl) is used instead of TBS buffer.

### Fluorescent labeling of fibronectin and 70-kDa fragment

Fibronectin or N-terminal 70-kDa fragment was labeled with Rhodamine Red™-X (FluoReporter Rhodamine Red™-X (RX) Protein Labeling Kit, Invitrogen) according to the manufacturer's instructions with a slight modification. RX was dissolved in DMSO and diluted in 0.5 M carbonate buffer, pH. 9.5, to a final concentration of 0.5 mg/ml, and fibronectin or the 70-kDa fragment were at a concentration of 2 mg/ml. Alternatively, fibronectin or the fragment was labeled with fluorescein isothiocyanate (FITC) as described [24].

### Fluorescence microscopy

Labeled fibronectin or 70-kDa fragment, 9 or 2.7 µg/ml (approximately 40 nM), was added to the culture medium at the time of addition of fibronectin-null cells to cover slips coated with various proteins. After 4-hr incubation at 37°C, cells were washed thrice and fixed with 3.7% paraformaldehyde for 15 min, followed by washing with PBS. Alternatively, cells were allowed to adhere for 2 hr and then incubated for an additional 2 hr with fibronectin or 70-kDa fragment before washing and fixation, or adhered for 1 hr and incubated for an additional 3 hr. Results were reproducible for differently labeled fibronectin (Rhodamine- or FITC-labeled fibronectin) added for slightly different durations by three different scientists over 10 year's period. The difference between different substrates within each figure was always consistent, although differently labeled fibronectin caused some difference between different figures. In other experiments, cells were also incubated with or without 500 nM lysophosphatidic acid (LPA) or 10 µg/ml 9EG7 (anti-mouse β1 integrin, Pharmingen, CA) during incubation. For dual fluorescence of FITC-fibronectin and rhodamine labeled actin, vinculin, or β1 integrin, cells were fixed and permeabilized with 3.7% paraformaldehyde for 15 min and 0.2% Triton X-100 for 5 min, using Rhodamine-phalloidin (Sigma, St. Louis, MO), monoclonal antibody against vinculin (Sigma v-9131), or 9EG7 against ß1 integrin followed by RX conjugated AffiniPure donkey anti-mouse or goat anti-rat IgG (Jackson ImmunoResearch).

Cover slips were mounted on Vectashield (Vector laboratories, Burlingame, CA) and viewed on an Olympus epifluorescence microscope (BX60, Olympus America Inc., Melville, NY). Pictures were taken with RT Slider digital camera (Spot Diagnostic Instruments, Inc., Sterling Heights, MI) and processed with Spot RT Software v3 and Adobe Photoshop (Adobe System Inc., San Jose, CA).

### Quantification of bound or deposited fibronectin, bound 70-kDa fragment, and other proteins

After incubation of fibronectin-null cells in DMEM containing 0.2% BSA and 40 nM RX-fibronectin, FITC-fibronectin, or FITC-70-kDa fragment at 37°C in wells coated with various proteins, cells were lysed with SDS buffer (1% SDS, 50 mM Tris, pH 7.4, 300 mM NaCl, 5 mM EDTA, 1 mM phenylmethyl sulfonyl fluoride, and protease inhibitor cocktail). The lysate proteins were separated by polyacrylamide gel electrophoresis in SDS (SDS-PAGE) and transferred to PVDF membrane (polyvinylidene fluoride, Millipore, Bedford, MA). RX- or FITC-labeled proteins were immunoblotted with rabbit anti-tetramethly rhodamine (Invitrogen) or anti-FITC as the primary antibody. Phosphorylated FAK Tyr-397 were immunoblotted with Anti-FAK [pY^397^] (Biosource, CA). Rabbit anti-actin (beta and gamma) rabbit polyclonal antibodies or anti-beta actin rabbit polyclonal antibodies (Abcam, Inc. Cambridge, MA), were also added to allow quantification of a “housekeeping protein” and normalization of results to amount of lysed cells. Bound primary antibodies were detected with peroxidase-conjugated donkey anti-rabbit IgG (Jackson ImmunoResearch, West Grove, PA), chemiluminescence reagent (Western Lightning™, PerkinElmer Life Sciences, Boston, MA), and Kodak® X-Omat AR film (Rochester, NY). Film was scanned (ScanJet 6200C, Hewlett Packard, Mountain View, CA), and band density was analyzed by Scion Image (Scion Corporation, Frederick, MD).

In some experiments, 1% deoxycholate (DOC) plus protease inhibitors [39] were added to the wells for 5 min, after which the DOC-soluble material was removed by careful aspiration. Fibronectin remaining after the extraction was then solubilized with SDS buffer and quantified by immunoblotting as described above. Normalization of DOC-insoluble material was accomplished by immunoblotting of actin in cells in replicate wells that were lysed with SDS without prior extraction.

## Results

### Fibronectin-null cells assemble exogenous fibronectin when adherent to 160/180 kDa tryptic fibronectin fragments or “fingerless” fibronectin, but not when adherent to ^7^F3-^14^F3 or shorter stretches of type III modules

**Figure 2:**
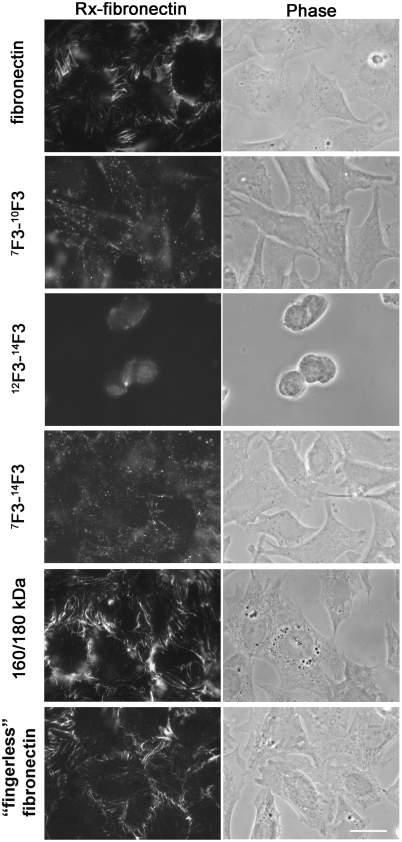
Fibronectin-null cells assemble exogenous fibronectin when adherent to 160/180 kDa fragments or “fingerless” fibronectin, but not when adherent to ^7^F3-^14^F3 or smaller type III-module containing proteins. Fibronectin-null cells in DMEM containing 0.2% BSA and 9 µg/ml RX-fibronectin were incubated at 37°C for 4 hr on cover slips coated with fibronectin, ^7^F3-^10^F3, ^12^F3-^14^F3, ^7^F3-^14^F3, 160/180-kDa fragments (160/180 kDa), or “fingerless” Fibronectin; at 3 µg/ml. Cells were fixed with paraformaldehyde and observed under the fluorescence microscope. The result is representative of 3 sets of experiments. Bar = 20 µm.

Because assembly of fibronectin is intimately related to the organization of the intracellular cytoskeleton [40]–[42], our original hypothesis for why fibronectin-null cells assemble exogenous fibronectin when adherent to a substrate coated with intact fibronectin but not when adherent to the cell-binding domain of fibronectin (modules ^7^F3-^10^F3) was that the heparin-binding HepII domain (modules ^12^F3-^14^F3), which enhances formation of actin-containing stress fibers in fibronectin-null cells [22], works alongside modules ^7^F3-^10^F3. To test the hypothesis, ^12^F3-^14^F3, ^7^F3-^10^F3, ^7^F3-^14^F3, or ^7^F3-^14^F3A was tested as substrates. Fibronectin-null cells adhered well to surfaces coated with all except ^12^F3-^14^F3, but after adherence the cells still failed to assemble exogenous RX-fibronectin (Fig. 2 and results not shown). As ^7^F3-^14^F3 contains the cell-binding and heparin-binding domains, we concluded that the presence of these integrin- and proteoglycan-binding domains in adsorbed pieces of fibronectin is not sufficient to support fibronectin assembly by adherent fibronectin-null cells.

To explore more broadly for the active region(s), proteolytic fragments of plasma fibronectin were tested as adhesive substrates. A coating of the gelatin-binding 160/180 kDa fragments, produced by limited trypsin digestion of native fibronectin, supported assembly very similarly to assembly supported by a coating of intact fibronectin (Fig. 2). As depicted in Fig. 1A, this mixture has 2 fragments in approximately equal amounts that each lack ^1^F1-^5^F1 and are monomeric due to cleavages that remove the C-terminal inter-chain disulfides [24]. The 160-kDa fragment also lacks a 31-kDa C-terminal segment containing modules ^15^F3 and ^10^F1-^12^F1 due to tryptic cleavage within the V region [43], whereas the 180-kDa fragment lacks the V region but includes the 31-kDa C-terminal extension containing ^15^F3 and ^10^F1-^12^F1; both fragments have the gelatin-binding domain and 14 type III modules [44] (Fig. 1A). Finding activity in the 160/180-kDa fragments demonstrated that modules ^1^F1-^5^F1, the extreme C-terminal tail, and a dimeric configuration due to the inter-subunit disulfides are not required for supportive activity.

**Figure 3:**
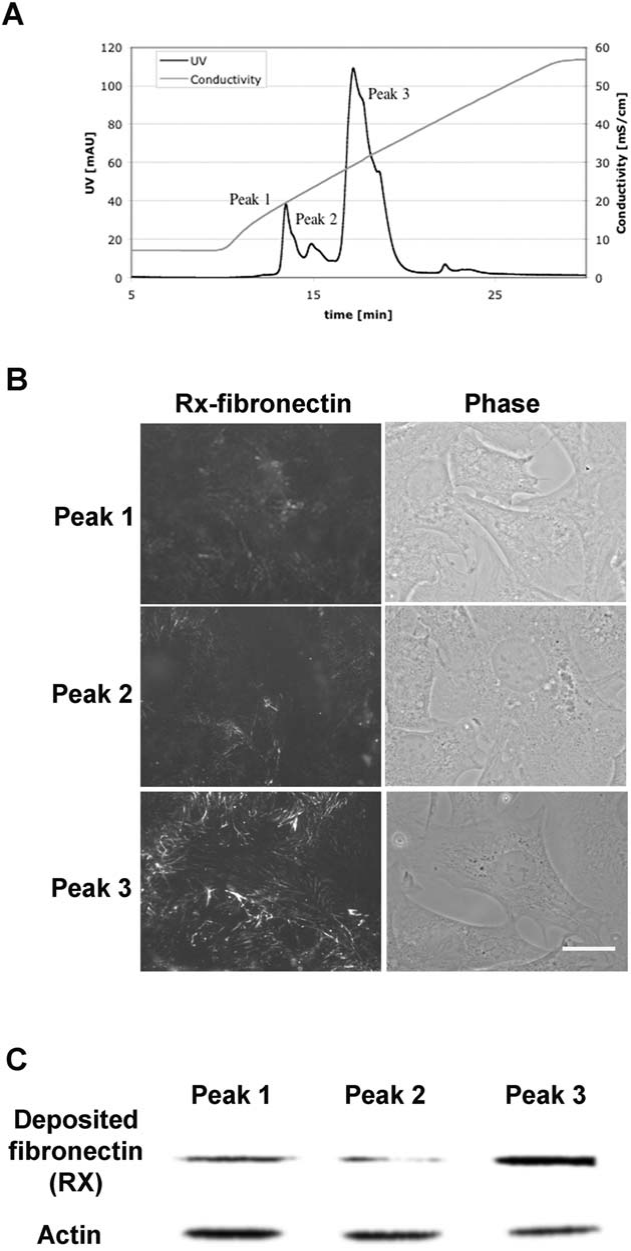
Identification of the fraction of “fingerless” fibronectin that supports exogenous fibronectin assembly by fibronectin-null cells as an adherent substrate. (A) “Fingerless” fibronectin was separated on a monoQ anionic exchange column. Three major peaks, 1, 2, and 3, were separated by a concentration gradient of NaCl. The likely modular compositions of fibronectin fragments in these peaks based on size and presence of epitopes for monoclonal antibodies are diagrammed in Fig. 1A and discussed in the text. (B) Supportive activity of the separated “fingerless” fibronectin proteins. After 2 hr incubation at 37°C of fibronectin-null cells on cover slips coated with peak 1, 2, or 3 at 3 µg/ml, 9 µg/ml Rx-fibronectin were added to culture medium, and cells were incubated for an additional 2 hr. Cells were assessed by fluorescence microscopy as described for Fig. 2. Bar = 20 µm. (C) After 4 hr incubation of fibronectin-null cells with 9 µg/ml Rx-fibronectin at 37°C on cover slips coated with Peak 1, 2, or 3 protein, 3 µg/ml, cells were lysed with buffer containing 1% SDS, anti-tetramethly rhodamine IgG fraction and anti-actin (beta and gamma) polyclonal antibody were used for detecting deposited labeled fibronectin and loading control, respectively. All panels are representative of at least 3 sets of experiments.

So-called “fingerless” fragments, a limited tryptic digest of reduced and alkylated fibronectin produced by a method intended to leave only the trypsin-resistant type III modules intact [25], also were active in supporting fibronectin assembly (Fig. 2). The fragment preparation contained a mixture of 180- to 200-kDa fragments when analyzed by SDS-PAGE (results not shown). These could be separated by FPLC (Fig. 3A). The 200-kDa fragment was found in Peak 3, and the smaller fragments were in Peaks 1 and 2 (results not shown). Fragments from all three peaks supported cell adhesion, but only a substrate coated with Peak 3 fragments supported assembly of fibronectin by adherent fibronectin-null cells (Fig. 3B and 3C). The compositions of the separated fragments were therefore determined by immunoblotting with monoclonal antibodies (results summarized in Fig. 1A). Peak 3 fragments contained epitopes for L8 and 9D2, which recognize ^9^F1-^1^F3 and ^1^F3, respectively [26], whereas Peak 1 and 2 fragments lacked the epitopes. IST-7, which recognizes ^15^F3 [26], detected proteins in Peaks 2 and 3. C4B10, which recognizes ^12^F3-^14^F3 [26], detected the protein in all three peaks. Peak 3 fragments were also recognized by monoclonal antibody 5C3 but not by 4D1. Antibody 5C3 recognizes an epitope in ^7^F1-^9^F1, whereas 4D1 recognizes an epitope in ^1^F1-^3^F1 (unpublished data). The 5C3 and L8 epitopes were destroyed by reduction of Peak 3 fragments with β-mercaptoethanol (results not shown). Approximately 50% of the protein in Peak 3 was found to bind to gelatin-agarose. These results, therefore, were interpreted as showing that a portion of Peak 3 fragments contains ^1^F3 and sequences from the gelatin-binding domain whereas Peak 1 and 2 fragments do not. The bands were diffuse even after purification (result not shown), which is compatible with size heterogeneity within each fraction. Further, binding of Peak 3 to gelatin and its recognition by L8 and 5C3, which occur only under non-reduced conditions [26], [45] (and results not shown), indicated that at least part of the gelatin-binding domain had not been completely reduced, alkylated, and digested during preparation of the fragments. The incomplete modifications fortuitously generated informative fragments that suggested that ^1^F3 and perhaps ^7^F1-^9^F1 of the gelatin-binding domain are necessary for the supportive activity of surface-adsorbed fibronectin fragments on fibronectin assembly.

### ^1^F3 and C-terminal modules are necessary for optimal supportive activity

**Figure 4:**
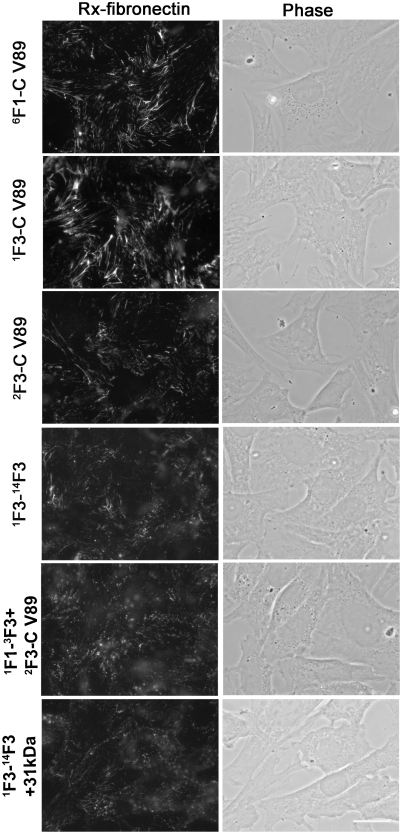
^1^F3 and the C-terminal segment are important and need to be on the same molecule for substrate activity. Assembly of RX-fibronectin by fibronectin-null cells adherent to ^6^F1-C V89, ^1^F3-C V89, ^2^F3-C V89, ^1^F3-^14^F3, ^1^F1-^3^F3+^2^F3-C V89, or ^1^F3-^14^F3+31 kDa, 3 µg/ml, was assessed by fluorescence microscopy as described for Fig. 2. The result is representative of 3 sets of experiments. Bar = 20 µm.

To define the contribution of the gelatin-binding domain and modules ^7^F1-^9^F1, ^1^F3, ^15^F3, and ^10^F1-^12^F1; recombinant ^6^F1-C V89, ^6^F1-CA V89, ^1^F3-C V89, ^1^F3-CA V89, ^1^F3-^14^F3, ^2^F3-^14^F3, and ^2^F3-C V89 were purified as secreted proteins after baculovirally directed expression (Fig. 1B) and tested as adherent substrates for fibronectin-null cells. The cells assembled exogenous fibronectin equally well when adherent to ^6^F1-C V89, ^6^F1-CA V89, ^1^F3-C V89, or ^1^F3-CA V89 (Fig. 4 and results not shown). These results, therefore, demonstrated that the gelatin-binding domain or ^7^F1-^9^F1 is not required for supportive activity.

**Figure 5:**
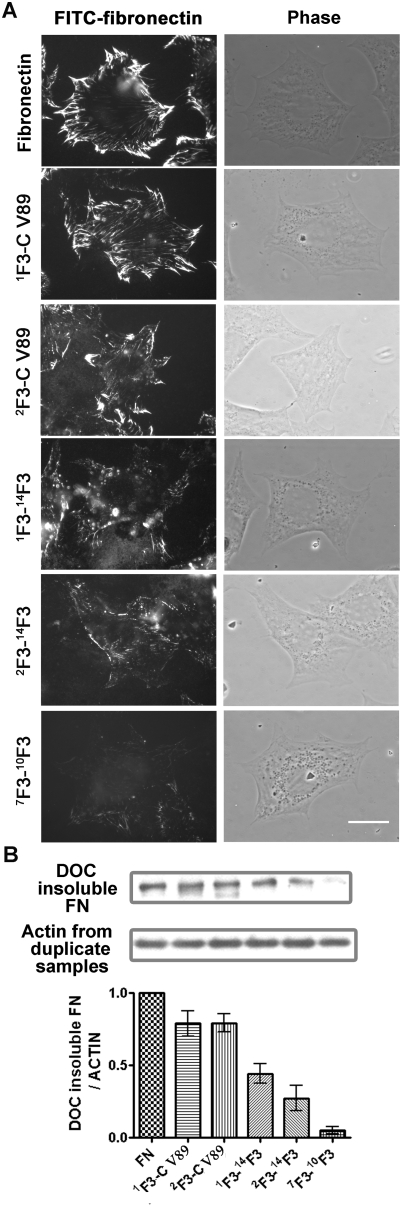
^1^F3 and C-terminal modules enhance supportive activity of fibronectin constructs. Fibronectin (FN) or recombinant fibronectin constructs, 20 nM, were coated on surfaces of coverslips or 24-well tissue culture plates. After 1 hr incubation at 37°C of fibronectin-null cells on the above proteins, 9 µg/ml FITC-fibronectin was added to culture medium, and cells were incubated for an additional 3 hr. (A) Cells were assessed by fluorescence microscopy as described for Fig. 2. Bar = 20 µm. (B) Assembly of FITC-fibronectin was assayed by immunoblotting after SDS-PAGE of material left after 5 min extraction with 1% deoxycholate (DOC insoluble FN). Anti-FITC and anti-beta actin polyclonal antibody were used for detecting deposited labeled fibronectin and loading control, respectively. The amount of DOC-insoluble fibronectin was normalized with actin in the total extract using duplicate samples and the relative amount is shown as bar graph. Each bar represents mean with standard error, 3 sets of experiments with duplicates in each (n = 6).

Fibronectin-null cells adherent to ^1^F3-C V89, ^2^F3-C V89, or ^1^F3-^14^F3 all bound FITC- or Rx-labeled fibronectin, but the fluorescent microscopic images of the bound protein were different for cells on ^1^F3-C V89 compared to cells on ^1^F3-^14^F3 or ^2^F3-C V89 (Fig 4 and Fig 5A). Fibrils associated with cells on ^1^F3-C V89 were numerous and aligned in an organized manner perpendicular to the edge of the cell. In contrast, cells on ^2^F3-C V89 or ^1^F3-^14^F3 bound fibronectin in fibrils that were less numerous, less elongated, and poorly organized.

Proteolytic fibronectin fragments and/or recombinant proteins were mixed together and used as substrates for culture of fibronectin-null cells to test if combinations of proteins containing ^1^F3 or the C-terminal modules are as effective as when both determinants of activity are present in the same protein. Co-coating of ^1^F1-^3^F3 with ^2^F3-C V89 or of ^1^F3-^14^F3 with 31-kDa fragment supported fibronectin assembly only minimally more than substrate coated with ^2^F3-C V89 or ^1^F3-^14^F3 alone (Fig. 4). A mix of recombinant ^1^F1-^3^F3, recombinant ^2^F3-^14^F3, and the 31-kDa tryptic fragment that contains ^15^F3 and ^10^F1-^12^F1 also poorly supported assembly (results not shown).

When the amount of fibronectin present in the deoxycholate-insoluble ECM pool was quantified, more fibronectin was assembled by cells on ^1^F3-C V89 or ^2^F3-C V89 than by cells on ^1^F3-^14^F3 or ^2^F3-^14^F3 (P<0.01) (Fig. 5B). These results, along with the supportive activities of Peak 3 “fingerless” fragments and the mix of 160- and 180-kDa tryptic fragments, provide evidence that ^1^F3 and the C-terminal modules are needed for optimal activity.

### Requirements for binding of the 70-kDa N-terminal fragment of fibronectin to cell surface sites of fibronectin assembly are the same as for assembly of intact fibronectin

**Figure 6:**
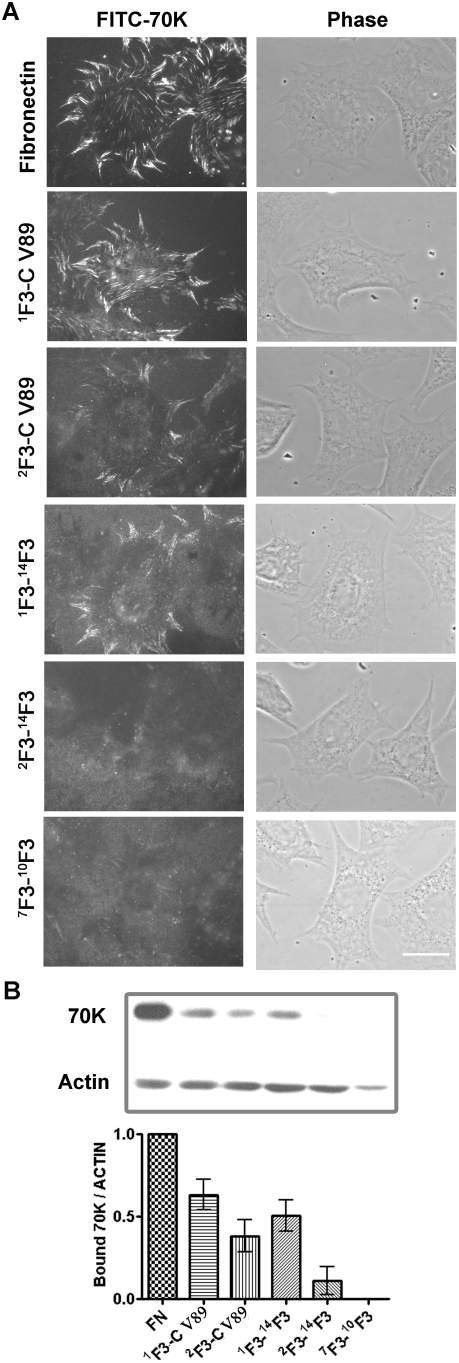
Binding of the 70-kDa N-terminal fibronectin fragment has the same requirement of adherent substrates. Fibronectin (FN) or recombinant fibronectin constructs, 20 nM, were coated on surfaces of coverslips or 24-well tissue culture plates. After 1 hr incubation at 37°C of fibronectin-null cells on the above proteins, 2.7 µg/ml FITC-70-kDa fragment was added to culture medium, and cells were incubated for an additional 3 hr. (A) Cells were assessed by fluorescence microscopy as described for Fig. 2. Bar = 20 µm. The result is representative of 3 sets of experiments. (B) Binding of FITC-70-kDa fragment was assayed by immunoblotting after SDS-PAGE of total extracts. Anti-FITC and anti-beta actin polyclonal antibody were used for detecting bound FITC labled 70-kDa fragment and loading control, respectively. The bar graphs represent relative amounts of bound FITC-70 kDa fragment in proportion to fibronectin as adherent substrate after normalization with actin in the same extract and were compiled from 3 experiments as in Fig. 5B (n = 6).

Binding of the N-terminal 70-kDa fragment of fibronectin to fibroblasts blocks assembly of intact fibronectin [24] and is an accurate indicator of the ability of various manipulations to enhance or diminish fibronectin assembly [11]. When FITC-labeled 70-kDa fragment was incubated with fibronectin-null cells adherent to fibronectin, ^1^F3-C V89, ^2^F3-C V89, ^1^F3-^14^F3, ^2^F3-^14^F3, or ^7^F3-^10^F3; fluorescence microscopy revealed patterns of deposition similar to the patterns of deposition of intact fibronectin (Fig. 6A, compare Fig. 6A with Fig. 5A). No binding was seen with cells adherent to ^7^F3-^10^F3 or ^2^F3-^14^F3; binding occurred with cells adherent to ^1^F3-C V89, ^1^F3-^14^F3, or ^2^F3-C V89; and a substrate of ^1^F3-C V89 best mimicked the patterns of binding exhibited by cells adherent to fibronectin. Western blotting demonstrated a hierarchy of binding, greatest for cells adherent to fibronectin and less for cells adherent to ^1^F3-C V89, ^2^F3-C V89, or ^1^F3-^14^F3 (Fig. 6B).

### V region, ^15^F3, and ^10^F1 are not required for support of fibronectin assembly

**Figure 7:**
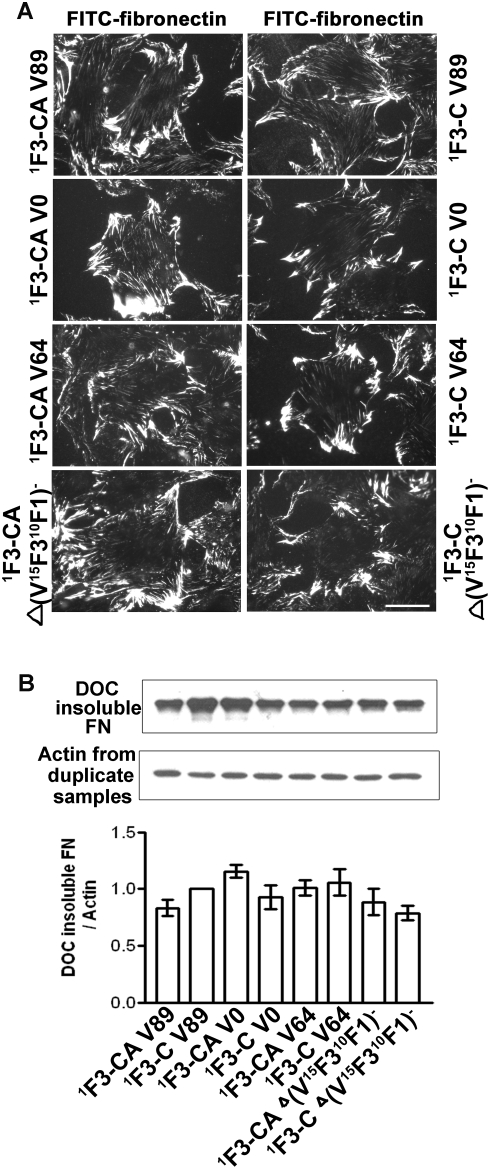
V region, ^15^F3, and ^10^F1 were not required for support of fibronectin assembly. ^1^F3-C V89 or the indicated other recombinant fibronectin constructs, 20 nM, were coated on surfaces of cover slips in 24-well tissue culture plates. After 1 hr incubation at 37°C of fibronectin-null cells on the above proteins, 9 µg/ml FITC-fibronectin was added to culture medium, and cells were incubated for an additional 3 hr. (A) Cells were assessed by fluorescence microscopy as described for Fig. 2. Bar = 20 µm. The result is representative of 3 sets of experiments. (B) Assembly of FITC-fibronectin was assayed by immunoblotting as described for Fig. 5B, 3 sets of duplicate experiments (n = 6).

To explore the important modules within the C-terminus, recombinant ^1^F3-C with alternatively spliced forms of the V region (V0, V64, Δ(V^15^F3^10^F1)) were generated with or without the alternatively spliced ^A^F3 module (Fig. 1C). Constructs ^1^F3-C V0/^1^F3-CA V0 (lacking the V region), ^1^F3-C V64/^1^F3-C V64 (contains the V64 version of V region instead of V89 version), and ^1^F3-C Δ(V^15^F3^10^F1)/^1^F3-CA Δ(V^15^F3^10^F1) (lacking V region, ^15^F3, and ^10^F1) all supported fibronectin assembly by fibronectin-null cells at the same level (Fig. 7), suggesting that V region, ^15^F3, and ^10^F1 are not required for support of fibronectin assembly and a variety of sequences may be present immediately C-terminal to ^12^F3-^14^F3. Similar results were obtained in 70K binding experiments (results not shown).

### Vinculin-containing focal adhesions co-localize with assembled fibronectin on supportive substrates but are also present on non-supportive substrates

**Figure 8:**
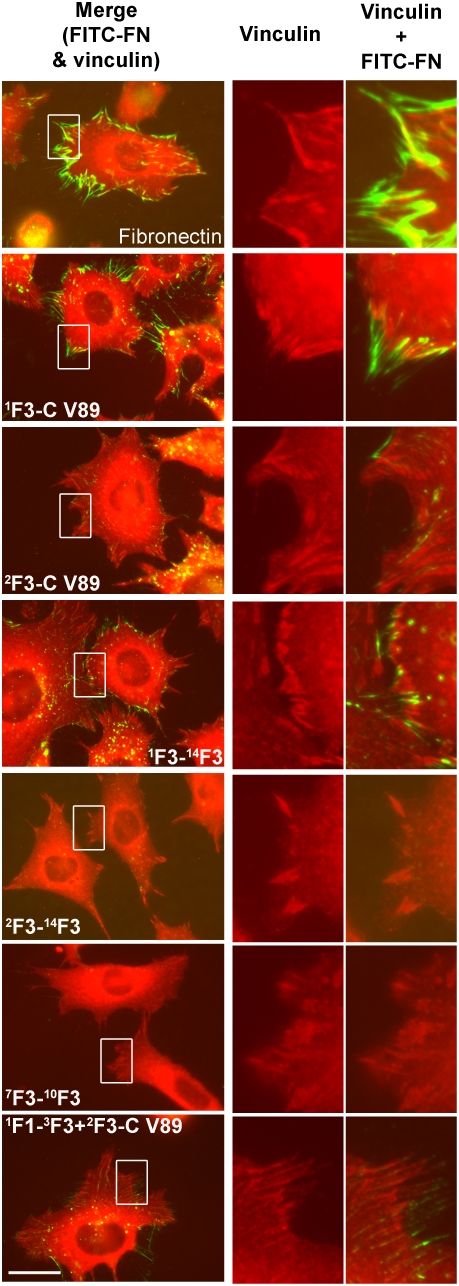
Vinculin co-localizes with assembled fibronectin on supportive substrates. Fibronectin or the indicated recombinant fibronectin constructs, 20 nM, were coated on surfaces of cover slips in 24-well tissue culture plates. After 1 hr incubation of fibronectin-null cells at 37°C on adherent substrates, 9 µg/ml FITC-fibronectin was added to culture medium, and cells were incubated for an additional 3 hr. Cells were assessed by fluorescence microscopy as described for Fig. 2. Panels under “Merge (FITC-FN & vinculin)” were merged images of vinculin (red) and FITC-FN (green). Panels under “Vinculin” or “Vinculin+FITC-FN” represent 4-fold enlargement of the white rectangular box. Bar = 20 µm. The result is representative of 2 sets of experiments.

To find out the relationship between assembled fibronectin fibrils and focal adhesions, dual fluorescence microscopy of assembled exogenous FITC-labeled fibronectin fibrils and vinculin stained by monoclonal antibody against vinculin and a Rhodamine-labeled secondary antibody was performed on fibronectin-null cells adherent to various substrates. Cells adherent to fibronectin, ^1^F3-C V89, ^2^F3-C V89, ^1^F3-^14^F3, ^2^F3-^14^F3, ^7^F3-^10^F3, or ^1^F1-^3^F3+^2^F3-C V89 all showed localization of vinculin in focal adhesions near the edges of cells (Fig. 8). The increased amount of FITC-Fibronectin assembled by cells adherent to fibronectin or ^1^F3-C V89 as compared to cells adherent to ^2^F3-C V89, ^1^F3-^14^F3, or ^1^F1-^3^F3+^2^F3-C V89 co-localized with coalesced vinculin (Fig. 8). However, similar patterns of vinculin-containing focal contacts were present in fibronectin-null cells plated on all constructs, including cells on ^2^F3-^14^F3 or ^7^F3-^10^F3 that completely lacked assembled FITC-fibronectin (Fig. 8).

### Explorations of mechanisms

The localization of vinculin into focal contacts of both fibronectin-assembly competent and incompetent cells raised questions of whether incompetency can be overcome. LPA is a stimulator of fibronectin assembly, which activates Rho and induces actin stress fiber formation [46]. Fibronectin-null cells adherent to ^2^F3-^14^F3 showed an increase in fibronectin assembly when stimulated with 500 nM LPA; however, the increased assembly was much less than on ^1^F3-C V89 (results not shown). The same was true when cells were stimulated with 10 µg/ml 9EG7 which activates β1 integrins [47] or both LPA and 9EG7 together; fibronectin assembly by cells on ^2^F3-^14^F3 was increased, but the increase was much less than similarly treated cells on ^1^F3-C V89 (results not shown). 9EG7 was also used to stain active β1 integrins. Fibronectin-null cells adherent to ^1^F3-C V89 had more active β1 integrins localizing to the periphery of the cells than cells on ^2^F3-C V89, with assembled fibronectin fibrils co-localizing with active β1 integrins (results not shown). However, for cells on ^2^F3-^14^F3, which could not assemble fibronectin, strong 9EG7 staining occurred at focal adhesions (results not shown). Finally, we found that phosphorylation level of FAK Tyr-397 was the same for fibronectin-null cells adherent to ^1^F3-C V89 or ^2^F3-^14^F3, regardless of assembly time ranging from 15 min to 3 hr (results not shown). Taken together, these results indicate that formation of focal adhesions by interaction with an adhesive protein maybe necessary but it is not sufficient for fibronectin assembly.

## Discussion

In short-term assays, fibronectin-null cells assemble exogenous fibronectin when adherent to a substrate coated with intact fibronectin but not when adherent to fibronectin's cell-binding domain (modules ^7^F3-^10^F3) recognized by α5β1 and other integrins [12]. We hypothesized that interactions of the adherent cell with regions of adsorbed fibronectin outside of modules ^7^F3-^10^F3 are required for display of the cell surface binding sites that initiate fibronectin assembly. Coatings of various proteolytic fibronectin fragments or recombinant fibronectin segments, each containing ^7^F3-^10^F3 and flanking modules, were tested for effects on fibronectin assembly by adherent cells or binding of the N-terminal 70-kDa fragment to assembly sites on these cells. A coating of recombinant ^2^F3-^14^F3, which contains the ^12^F3-^14^F3 heparin-binding domain as well as the cell adhesion domain, was not effective in supporting fibronectin assembly. Addition of module ^1^F3 or C-terminal modules to modules ^2^F3-^14^F3 resulted in some activity, and addition of both ^1^F3 and the C-terminal modules resulted in a construct, ^1^F3-C, that approached the activity of intact fibronectin. Co-coating experiments revealed that the required modules work best when present in the same piece of fibronectin. The fact that ^1^F3-C V0, ^1^F3-C V64, and ^1^F3-C Δ(V^15^F3^10^F1) all supported fibronectin assembly indicates that the V region, ^15^F3, and ^10^F1 are not needed for supportive activity. These results suggest that ^1^F3 acts together with fibronectin C-terminal modules to cause adherent cells to assemble fibronectin. There was no requirement for two features that are necessary for soluble fibronectin to be assembled: the N-terminal 70-kDa portion, which directs soluble fibronectin to cell surface assembly sites [11], and a dimeric configuration, which presumably is required to perpetuate “head-to-tail” interactions between fibronectin subunits during fibril growth [48]. The grouping of fibronectin modules required for a substrate to support assembly of soluble fibronectin by adherent cells, therefore, is novel and different from the groupings required for optimal cell adhesion or for soluble fibronectin to bind to and become insolubilized at assembly sites.

Are the results due to differences in the conformation in which the purified fragments or recombinant proteins adsorb to surfaces? Pieces of fibronectin that failed to support fibronectin assembly strongly supported cell adhesion per se, demonstrating that the RGD sequence in ^10^F3 of the various adsorbed proteins is available to bind integrins. Similar composition/activity relationships were found for the set of proteolytic fragments and the set of recombinant proteins, even though the recombinant proteins all had the same N- and C-terminal “tails” introduced by the cloning strategy [38] as compared to the different and variable surrounding sequences in the fragments generated by limited tryptic digestion of native and of reduced and alkylated fibronectin. Similar effects were seen on glass cover slips and tissue culture dishes. Finally, in results not presented, we coated ^1^F3-C V89 or ^2^F3-^14^F3 in different coating concentration varying from 5 nM to 80 nM and detected the amount of protein adsorbed to the wells by ELISA using an antibody (MabIII10) recognizing the ^10^F3 module. ^2^F3-^14^F3 adsorbs similarly as ^1^F3-C V89 (results not shown), suggesting the difference in fibronectin assembly is not due to difference in protein adsorption when coating. Thus, the results were consistent despite changing variables that would be expected to influence conformations that proteins adopt upon adsorption to surfaces. We therefore favor the hypothesis that ^1^F3-C V89, the 160/180-kDa fragments, or the Peak 3 “fingerless” fragments imparts information to adhering cells that is missing in ^2^F3-^14^F3 or smaller pieces of fibronectin. Recognition of ^1^F3 and the C-terminal region by adhering cells results in a phenotype in which binding sites for the N-terminal region of soluble fibronectin are expressed at specific regions on the surface of the adherent cell.

^1^F3, along with ^2^F3, is known to influence fibronectin assembly in cells expressing fibronectin and is thought to work, at least in part, by mediating fibronectin-fibronectin interactions [26], [49]–[53]. Denaturation or mutagenesis of ^1^F3 exposes cryptic sites for association with other parts of fibronectin [50], [54], [55] and formation of a ternary complex with heat-denatured ^10^F3 and the N-terminal 70-kDa fragment [50], [56]. ^1^F3 has also been shown to bind to ^7^F3 and ^15^F3 [54]. Treatment of purified fibronectin in solution with a recombinant protein that contains ^1^F3 lacking the N-terminal part of the module results in multimerization of fibronectin [51], [57]. The truncated ^1^F3 binds to modules ^1^F3-^3^F3 and ^11^F3, probably after a spontaneous opening of these modules [58]. Such spontaneous opening followed by domain swapping may be a key event in assembly of fibronectin fibrils [58]. Force–induced unfolding of ^1^F3 and other modules has been considered as a possible step in fibronectin fibril formation [59], and N-terminally truncated ^1^F3 has been suggested to mimic an intermediate in a simulated forced unfolding of the intact ^1^F3 module [60]. Nevertheless, recombinant rat fibronectin lacking either ^1^F3 or ^1^F3 through ^7^F3 is assembled into fibrils [49], [53]. Although these fibrils have some structural differences, this observation indicated that fibronectin-fibronectin interactions mediated by ^1^F3 are not essential for formation of fibronectin fibrils.

Identification of ^1^F3 as one of the regions in substrate-adsorbed fibronectin required for support of assembly of exogenous fibronectin by adherent cells represents a new activity of ^1^F3 in fibronectin assembly. Relating our finding to studies of mutated, truncated, or denatured ^1^F3 is difficult because ^1^F3-containing proteins in our experiment contained other type III modules, including ^3^F3, ^10^F3, and ^11^F3, and were present in the adhesive substratum rather than in solution. In addition, the activity of coatings of pieces of fibronectin containing ^1^F3 is mimicked by coatings of laminin [11], [12] or type I collagen [61], [62].

On fibronectin-null cells, fibril extension occurs on elongated focal adhesions near the cell periphery and the sites where 70K binds [11]. Cells formed similar looking (vinculin-stained) focal adhesions when plated on any of the fibronectin constructs tested. However, cells were only capable of matrix assembly and 70K binding when the construct contained ^1^F3 and the C terminus. One possibility is that there is an unknown receptor that recognizes ^1^F3 in this context and initiates a signaling pathway that activates matrix assembly. Mechanisms for the action of ^1^F3 have been proposed that involve direct interactions with cells [63]–[65]. We speculate, therefore, that ^1^F3 and unknown regions of laminin and type I collagen engage receptors on adherent cells, generating signals that make the cells competent to assemble fibronectin. Laminin or type I collagen may not necessarily interact with the receptor(s) that recognize(s) ^1^F3 of fibronectin.

Identified receptors in the C-terminal part of fibronectin include α4β1 [66], [67], syndecan proteoglycans 68, 69, and RGD-independent α5β1 interaction induced by the urokinase plasminogen activator receptor (uPAR) [70], [71]. We could not find evidence that integrin α4β1 is an adhesion receptor in the fibronectin-null cells used in the present experiments, i.e., the cells did not adhere to a coating of vascular cell adhesion molecule (VCAM) (results not shown). Syndecans and uPAR are known to influence fibronectin assembly by fibroblasts and bind to ^12^F3-^14^F3 or ^12^F3-^15^F3 [70]–[72], respectively. Syndecans act as co-receptors to modulate integrin-mediated cell–matrix adhesion [73], and distinct signal-transduction events appear to follow syndecan ligation by heparin-binding sites in matrix molecules [22]. CHO cells that lack glycosaminoglycan synthesis poorly assemble fibronectin [74]. Cells lacking syndecan-2 are deficient in α5β1-mediated attachment to fibronectin [75], and cells expressing syndecan-2 without the C-terminal 14 amino acids of the cytoplasmic domain do not assemble fibronectin into a fibrillar matrix [72]. The effect of uPAR on fibronectin cell adhesion also appears to involve α5β1, so that α5β1 recognizes a site in the ^12^F3-^15^F3 region under the influence of uPAR [70].

Remarkably, vitronectin, which suppresses fibronectin assembly by adherent cells [9], [12]–[14], engages the same sorts of receptors as fibronectin, i.e., RGD-recognizing integrins, proteoglycans [13], and uPAR [76], and cell adhesion to vitronectin is enhanced by interactions among these receptors [76]–[78]. One way to explain the suppressive activity of vitronectin is to extend the line of reasoning that seeks to explain supportive activity of adhesive ligands, hypothesizing that a suppressive adhesive ligand may engage a similar set of receptors as are engaged by a supportive ligand but with opposite results. Another possibility is that vitronectin suppression is related to findings that 70K may bind αvβ3 integrin and initiate fibronectin matrix assembly in the absence of fibronectin's RGD [10]. αvβ3 may be activated by the bound 70K or fibronectin and track to the focal adhesion [79]. Thus, if αvβ3 is, as suggested [10], sequestered by interactions with adsorbed vitronectin, the integrins would not be available to perform the tracking function. One must then explain why cells adherent to laminin, collagen I, and ^1^F3-C are able to accomplish for this recruitment, whereas cells adherent to fibronectin constructs lacking ^1^F3 and the C-terminal modules are inactive. At present, this remains an enigma.
